# Partisan Stability During Turbulent Times: Evidence from Three American Panel Surveys

**DOI:** 10.1007/s11109-022-09825-y

**Published:** 2022-11-25

**Authors:** Donald P. Green, Paul Platzman

**Affiliations:** Department of Political Science, Columbia University, 7th Floor, International Affairs Building 410 W. 116th Street, New York, NY 10027 USA

**Keywords:** Party identification, Public opinion, Panel surveys

## Abstract

The past decade has witnessed profound changes in the tenor of American party politics. These changes, in tandem with growing affective polarization and residential segregation by party, raise the question of whether party identification is itself changing. Using three multi-wave panel surveys that stretch from the first Obama Administration through the Trump Administration, this paper takes a fresh look at the stability of party identification, using several different statistical approaches to differentiate true partisan change from response error. Perhaps surprisingly, the pace of partisan change observed between 2011 and 2020 is quite similar to the apparent rates of change in panel surveys dating back to the 1950s. Few respondents experience appreciable change in party identification in the short run, but the pace at which partisanship changes implies that substantial changes are relatively common over a voter’s lifespan.

**Supplementary Information:**

The online version contains supplementary material available at 10.1007/s11109-022-09825-y.

## Introduction

Recent years have witnessed growing partisan division. The phenomenon of affective polarization, whereby partisans increasingly dislike their partisan rivals, seems to have accelerated after 2000 (Iyengar, 2022). According to Mason (2018), partisans themselves have become more “sorted” insofar as Democrats increasingly identify as liberal and Republicans increasingly identify as conservative, a pattern she argues amplifies these groups’ emotional investment in politics. At the same time, opposing partisans have become more geographically (Brown & Enos, 2021) and socially (Webster et al., 2022) segregated, a pattern that may facilitate the transmission and expression of partisan attachments. Whether these underlying trends in mass partisanship reflect elite cues or create incentives for partisan invective among party leaders, public officials of both parties have increasingly expressed their contempt for their partisan opponents (Fowler et al., 2016; Parker, 2020). The current political environment, described as “turbulent” by contemporary historians (Zelizer, 2022), makes the party politics of the Reagan era seem genteel.

At the same time, the parties themselves have changed. The advent of Donald Trump brought about profound changes in the Republican Party’s platform (Rubin, 2016), and his pugnacious nativism and disdain for political convention attracted new support among non-college educated Whites (Vavreck, 2020). Reflecting on the changes he brought about, Trump commented that “The party is a much bigger party now, and it’s like really a party for the working men and women in this country, in addition to plenty of others...In the true sense, it has been changed” (Bennett, 2018). Trump’s transformation of the GOP has become increasingly prominent in academic commentary. Gary Jacobson, for example, argues that “By projecting a sharp, highly divisive image of who and what the Republican Party stands for and, equally important, who and what it stands against, the Trump presidency is poised to have a durable impact on party images and identities, especially among younger citizens entering the electorate for the first time” (Jacobson, 2018). On the Democratic side, the Progressive wing’s policy proposals and leaders rose to new prominence (Raphael and Todd, 2016) with the presidential primary bid of Bernie Sanders in 2016 and the meteoric rise of Alexandria Ocasio-Cortez in 2018. As Zingher (2018) notes, “In the aggregate, white citizens have viewed the Democratic Party as moving progressively further and further away from their own position. An increasing majority of whites hold the perception that the Democratic Party has followed the shifting median on the economic dimension to the left” (p. 870).

The changing issue stances and social imagery of the parties raise the question of whether events of recent years have led voters to rethink their fit with the parties. Has the tumult of contemporary party politics hastened the pace with which party identities change, as disaffected issue voters gravitate away from their former party? Or, conversely, have the new features of party politics contributed to more stable party ties that are buttressed by more ardent emotions and perceived social distance between warring partisan camps?

This paper revisits the decades-old research literature that uses multi-wave panel surveys to assess the extent to which respondents’ party attachments change over time. Studies of this kind date back to *The American Voter* (Campbell et al., 1960) but gained methodological sophistication in the wake of Asher (1974) and Achen (1975), which called attention to the ways in which response error may exaggerate the actual rate of partisan change. One corrective has been to use instrumental variables regression or similar maximum likelihood estimators to distinguish observed change from true change in latent party identification (Palmquist & Green, 1992). For example, Green and Palmquist (1994) apply measurement error models to nine multi-wave panel studies from the United States, and Schickler and Green (1997) do so for eight panel studies from outside the US. Their results, which show party identification to be highly stable but by no means immutable, provide a benchmark for comparison. However, modeling approaches of this kind are not without their critics, and other approaches have gained prominence as well. One such approach is to create additive indices based on multiple measures of an underlying factor and to assess the over-time correlations of these more reliable indices (Ansolabehere et al., 2008). More recent works, such as Tucker et al. (2019), offer modeling innovations of their own, capitalizing on the fact that internet panels often feature many more waves of interviews than traditional face-to-face surveys. To our knowledge, however, no one has attempted to pull together an assortment of contemporary panel surveys in order to assess whether methodologically robust conclusions may be drawn about the pace of partisan change in the current partisan environment.

Our essay is organized as follows. We begin by reviewing leading theories about the conditions under which party attachments change over time, connecting each to scholarly debates about the political implications of demographic, technological, and institutional change. By our reading, recent trends have ambiguous implications for partisan stability, as some theories suggest the potential for instability due to changes in party positions on salient issues, while others suggest the potential for stability on the grounds that the parties are more socially distinctive than before. Next, we introduce three multi-wave panel surveys that collectively cover the period 2011 to 2020. Before attempting to model patterns of partisan change, we provide both quantitative and graphical descriptions of how stated party attachments evolve over successive interviews. Measurement models are introduced and applied to the three panel surveys, and these results are cross-validated using other approaches, namely, the creation of multi-item indices and the use of regression to estimate the over-time trajectories of individual respondents. A consistent picture emerges from all of the approaches. Perhaps surprisingly, party attachments over the past decade have changed very gradually, and party identification in contemporary panel studies appears to be at least as stable as it was in the American National Election Studies panel surveys dating back to the 1950s. We conclude by spelling out the implications of these findings for long-term changes in partisanship. Although substantial changes in party identification are rare over the course of a single election campaign or presidential term, meaningful change is common over the span of a voter’s lifetime.

## Competing Perspectives on Partisan Stability

Although party attachments in the United States have long been characterized as unusually stable compared to other political attitudes (Converse, 1964), the importance of party identification as an explanatory construct has led generations of public opinion scholars to offer hypotheses about the conditions under which party attachments change. Propositions about why change may be afoot in the current era trace their roots to longstanding arguments that fall roughly into three theoretical categories.

The first concerns the “spatial” proximity between the voter and the parties’ positions on leading issues. This perspective (Franklin and Jackson, 1983; Jackson, 1975; Kollman & Jackson, 2021) contends that when new issues become prominent or when parties change their platforms, voters gravitate toward the party that is more congenial to their policy views. This theory directs our attention to two sources of change, the repositioning of the parties vis-à-vis issues and the ways that technological change may have drawn public attention to specific issues and where the parties stand on them.

There are good reasons to suppose that issue-based evaluations of the parties have grown increasingly important over time. The two major parties present far more ideologically distinctive “brands” than in decades past (Butler & Powell, 2014), and the ranks of prominent elected officials feature a dwindling number of conservative Democrats or liberal Republicans (Fiorina, 2017). Although public opinion scholars disagree about the extent to which the public has sorted itself into ideologically distinctive partisan camps (Abramowitz, 2018; Levendusky, 2009), as opposed to updating their issue stances to fall into line with the positions staked out by party leaders (Barber & Pope, 2019; Lenz, 2012), it seems clear that the correlation between party and self-described liberalism/conservatism has climbed over time (Bafumi & Shapiro, 2009). At the same time, the parties have staked out increasingly divergent positions on issues that were largely orthogonal to party prior to the 1990s, such as gun rights (Joslyn et al., 2017) and immigration (Fennelly et al., 2015), and the same may be said of high-salience moral issues that are sufficiently resonant with the public to upend their party attachments (Goren & Chapp, 2017). As noted above, the Trump era is noteworthy for its changes in Republican Party stances, and much the same could be said for the increasingly visible Progressive wing of the Democratic Party. For authors such as Kollman and Jackson (2021, Chap. 7), such shifts in issue stances have the potential to profoundly alter the balance of party identification.

On the other hand, public opinion scholars have long expressed skepticism about whether the public is sufficiently knowledgeable and concerned about issues to use spatial proximity when evaluating candidates (Stokes, 1963) or parties (Converse, 1964). Although this critique continues to find support in contemporary surveys (Kinder & Kalmoe, 2017), another school of thought contends that a growing segment of the American public does care about issues (Bullock, 2011; Carsey & Layman, 2006; Mummolo et al., 2021), and their ability to connect their issue stances to their party evaluations has been aided by the advent of ideologically polarized television networks (DellaVigna & Kaplan, 2007), social media (Cacciatore et al., 2016), and permissive campaign finance rules (Brooks & Murov, 2012) that together have greatly increased the volume of communication that voters receive about issues. On balance, the issue proximity hypothesis seems to imply that party affiliations should have changed at a faster-than-usual pace between 2011 to 2020.

Another prominent theory has to do with the social imagery of the two parties. What kinds of people come to mind when one thinks about Democrats or Republicans? The prodigious literature on social stereotypes and group identities (Green et al., 2002; Huddy et al., 2015) also finds expression in two competing hypotheses, one that emphasizes the potential for change and another, the hardening of existing party attachments. The former focuses on White identity politics amid Whites’ growing concern about ethnic change (Craig & Richeson, 2014; Jardina, 2019; Major et al., 2018). White ethnic appeals may be seen as a force that pulled Whites in the direction of the GOP during this period, especially after Obama’s election notified White voters that the Democratic Party is preferred by and supportive of Black Americans (Tesler & Sears, 2010). However, recent experimental studies call into question whether interventions that prime White ethnic concerns in fact make them more likely to embrace the Republican Party (McCarthy, 2022). By the same token, the widely-publicized criticisms of undocumented immigrants from Mexico by Trump and prominent GOP figures might have led to increasing Democratic attachment among Hispanics, although a series of experimental tests by Hopkins et al. (2020) suggest that such effects on partisan attachments are surprisingly weak. Perhaps driven by the normalization of “uncivil discourse” (Mutz, 2015) between politicians from warring camps, recent years have seen a surge in “affective polarization” by which partisans increasingly dislike one another (Druckman & Jeremy, 2022). Coupled with increasing residential and social segregation by party, the tenor of current politics may have helped to harden existing partisan social divisions. On balance, the social imagery hypothesis seems to suggest a slower-than-usual pace of partisan change during the 2011–2020 period.

Finally, performance evaluations have long figured prominently as explanations of individual-level (Fiorina, 1981) and aggregate-level (MacKuen et al., 1989) partisan change. The economic swings of the past decade, combined with a media landscape that accentuates economic resentment (Soroka, 2014), have led many to speculate about the extent to which disaffection with the one or both parties reflects the way in which they are blamed for the loss of jobs, stagnant wages, and rising personal debt. This line of argument is also used to explain the growing regional partisan divide between the more economically vibrant coasts and the country’s interior (Hopkins, 2017). The public’s favorable assessment of the national economy under Trump (Small & Eisinger, 2020) would have been expected to attract new Republican partisans, at least until the COVID-19 epidemic led to an abrupt economic contraction. However, Trump’s relatively low approval ratings throughout his term offset the partisan gains that might ordinarily follow from an economic surge. In the end, the performance records of neither Obama nor Trump are sufficiently distinctive to imply faster-than-usual rates of partisan change.

Taken together, these three theoretical perspectives offer competing empirical predictions. The character and pace of what Carmines and Stimson (1989) termed “issue evolution” militates in favor of a quickened pace of partisan change; on the other hand, the widening social divide between the two parties arguably makes partisans more resistant to change, while the vicissitudes of presidential performance seem to imply no clear prediction. We therefore consider the core hypothesis to be two-sided, given the strong theoretical reasons to suspect issue-driven change or affect-driven stability.

## Description of Current Studies

To our knowledge, there are three publicly available multi-wave panel surveys of American adults spanning the Obama to Trump administrations that repeatedly measured party identification: the Institute for the Study of Citizens and Politics (ISCAP) survey,^1^ Views of the Electorate Research Survey conducted by the Democracy Fund Voter Study Group (VSG),^2^ and The American Panel Survey (TAPS).^3^ The three panels started and finished at somewhat different times but collectively spanned November 2011 to October 2020.

Each of the three panel surveys utilized sampling procedures designed to recruit a nationally representative set of respondents. ISCAP panelists were recruited offline via address-based sampling or random-digit dialing, and panel demographics closely mirrored the adult population of the United States. VSG respondents were recruited according to a stratified sample that matched US population benchmarks. TAPS respondents were recruited via address-based sampling and drew upon a number of publicly available datasets, including US Census files, the White Pages, and credit agency data, to sample in proportion to US demographic benchmarks. In addition, all three panels employed survey weights after each survey wave to improve sample representativeness.

Each panel survey repeatedly measured party identification on a seven-point scale (PID-7) using the American National Election Study (ANES) wording, with slight variations.^4^ The main differences across panels have to do with the number of times respondents were interviewed. The ISCAP panel measured PID-7 on nine occasions,^5^ VSG six, and TAPS twenty-four.^6^ For compactness and comparability, many of the analyses below will draw from exactly six waves for each panel. For VSG, this represents the entire panel. For ISCAP and TAPS, this represents the first and last wave and four waves selected at regular intervals in between. (Analyses of the full ISCAP and TAPS panels are provided in the Online Appendix.) See Table 1 for the survey dates for each of the six waves selected for analysis from each panel; for the full ISCAP and TAPS panel wave dates, see Online Appendix Tables A1 and A2, respectively.

**Table 1:** Wave field dates and PID-7 responses per wave

	Start date	End date	PID responses
ISCAP			
Wave 6	10/19/2012	10/29/2012	2258
Prior to Wave 10$^{*}$	7/28/2015	9/3/2015	1382
Prior to Wave 11$^{*}$	7/15/2016	8/27/2016	1158
Wave 13	10/23/2018	11/5/2018	1007
Prior to Wave 14$^{*}$	6/11/2019	8/20/2019	926
Wave 15	10/7/2020	10/22/2020	1030
VSG			
Wave 1	December 2011		7827
Wave 2	After 2012 Election		7969
Wave 3	11/29/2016	12/29/2016	7971
Wave 4	7/13/2017	7/24/2017	5940
Wave 5	4/5/2018	5/14/2018	5847
Wave 6	11/17/2018	1/7/2019	6706

	Survey month		PID responses
TAPS			
Survey 1	November 2011		1271
Survey 11	October 2012		1417
Survey 25	December 2013		1377
Survey 38	January 2015		1460
Survey 54	May 2016		1555
Survey 70	January 2018		1941

$^{*}$Respondents whose PID response date was not within their wave’s start and end dates were recoded as missing

The panels also varied greatly in the number of respondents they interviewed; the right-hand column of Table 1 presents the number of respondents who completed the PID-7 survey items in each wave. VSG recruited considerably more respondents (often nearing 8000) at each survey wave than ISCAP or TAPS, which each averaged between one and two thousand respondents per wave. Due to a combination of budget limitations and respondent fatigue, only 365 ISCAP respondents, 4013 VSG respondents, and 445 TAPS respondents provided PID-7 survey responses for each of their panel’s waves (we refer to these respondents as “complete cases”). We consider the implications of panel attrition below. The rates of attrition undermine the justification for using sample weights; for simplicity, we present unweighted results, although as shown in Online Appendix Table A16 and Online Appendix Fig. A1, weighted estimates tend to be similar.

### Changes in Mean Partisanship over Time

Before delving into patterns of individual-level changes over time, we first characterize patterns of aggregate change. We do so in two ways. First, Table 2 reports the means and standard deviations of PID-7 for complete case panelists in each survey. The means move very subtly in the Republican direction over time. For example, VSG’s mean on a scale ranging from $-\,3$ to $+\,3$ was − 0.19 at the end of 2011 and − 0.15 approximately eight years later. We also observe a slight increase in standard deviation, consistent with the notion that partisan attachments strengthen with age (Achen, 1992).

**Table 2:** PID-7 wave-level metrics

	Start date	End date	Mean	SD
ISCAP (complete cases: N = 365)				
Wave 6	10/19/2012	10/29/2012	− 0.28	2.31
Prior to Wave 10	7/28/2015	9/3/2015	− 0.20	2.36
Prior to Wave 11	7/15/2016	8/27/2016	− 0.21	2.33
Wave 13	10/23/2018	11/5/2018	− 0.23	2.38
Prior to Wave 14	6/11/2019	8/20/2019	− 0.23	2.42
Wave 15	10/7/2020	10/22/2020	− 0.13	2.42
VSG (complete cases: N = 4013)				
Wave 1	December 2011		− 0.19	2.18
Wave 2	After 2012 Election		− 0.24	2.23
Wave 3	11/29/2016	12/29/2016	− 0.20	2.21
Wave 4	7/13/2017	7/24/2017	− 0.17	2.19
Wave 5	4/5/2018	5/14/2018	− 0.17	2.21
Wave 6	11/17/2018	1/7/2019	− 0.15	2.25

	Survey month		Mean	SD
TAPS (complete cases: N = 445)				
Survey 1	November 2011		− 0.13	2.15
Survey 11	October 2012		− 0.12	2.32
Survey 25	December 2013		− 0.13	2.21
Survey 38	January 2015		− 0.06	2.27
Survey 54	May 2016		− 0.11	2.27
Survey 70	January 2018		− 0.08	2.26

The PID-7 scale ranges from − 3 (“Strong Democrat”) to $+$ 3 (“Strong Republican”)

**Fig. 1:**
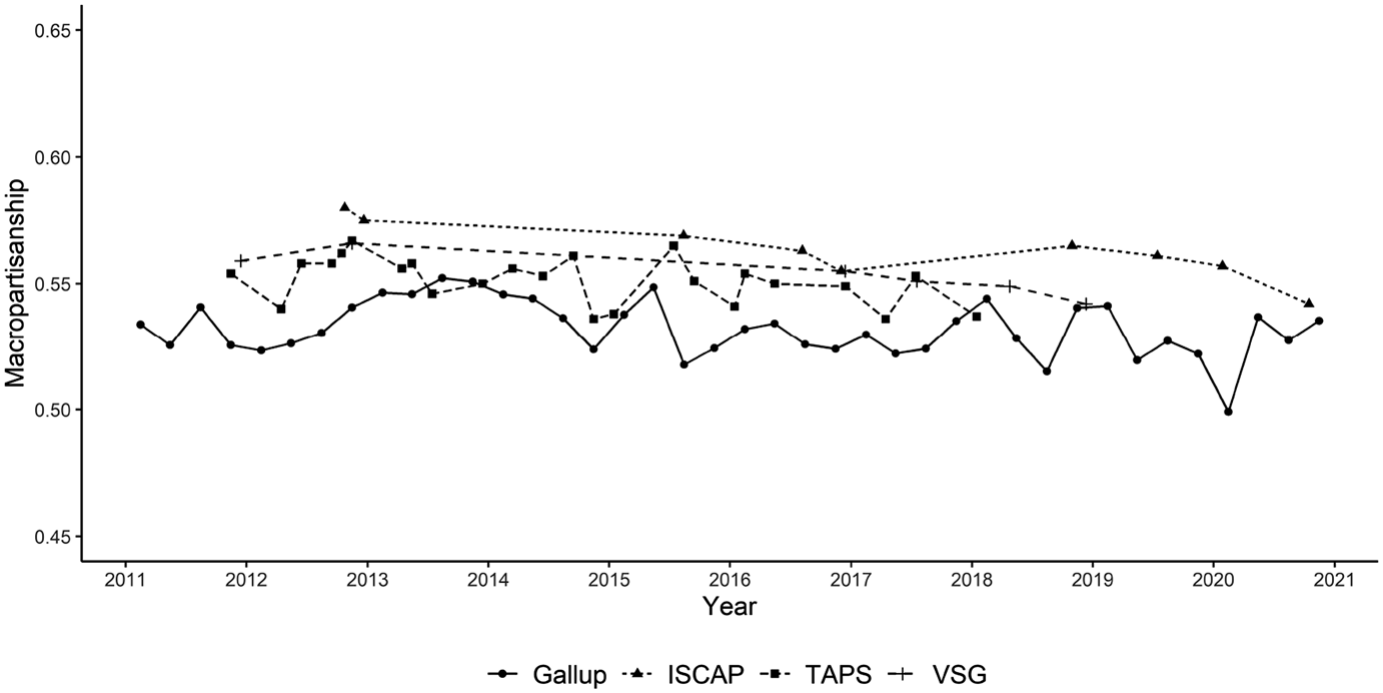
Measures of macropartisanship between 2011 and 2020 from quarterly Gallup polls and ISCAP, VSG, and TAPS panel survey waves

Second, Fig. 1 visualizes aggregate trends among complete case panelists. Following MacKuen et al. (1989), we code “macropartisanship” as the proportion of party identifiers (Democrats or Republicans, based on the 3-category stem question) who identify as Democrats. This coding is applied to each of the three panel studies, allowing us to track macropartisanship among the same respondents over time. For reference, the graph also displays quarterly macropartisanship readings compiled from cross-sectional Gallup surveys conducted over the same period.

The partisan balance in the American electorate changed very little over this time period. For each of the three panel surveys, macropartisanship scores are essentially flat, tilting slightly in the Republican direction. This pattern is not specific to panel respondents; the Gallup macropartisanship is also quite placid during this period. Whereas the quarterly macropartisanship series between 1953 and 1987 originally studied by MacKuen et al. (1989) had a standard deviation of 0.041, the standard deviation during the 2011–2020 period was only 0.011. The fact that aggregate trends look similar for both panel surveys and cross-sectional surveys suggests that panel attrition plays a relatively minor role in shaping the results. The next section specifically addresses attrition before analyzing individual-level dynamics.

### Panel Attrition

In order to explore the consequences of panel attrition, we sought to model the kinds of respondents who dropped out over time. This exercise was not straightforward—panelists may have discontinued their participation by choice or because they were not invited by survey administrators to continue their participation. Panel administrators reported facing resource constraints, prompting them to adapt their sampling methodology or alter subgroup quotas over time.^7^ Unfortunately, these decisions are not clearly documented in the accompanying meta data. Patterns of attrition are sometimes erratic—some respondents provided PID-7 responses in waves subsequent to waves in which they did not—complicating our ability to characterize a panelist’s status. Despite these limitations, we note that in each panel, the number of remaining complete cases monotonically declined across waves. Moreover, across the three panels, an average of 10.8% to 13.5% of all PID-7 response opportunities among respondents completing a given survey were missing. The similarity of these proportions suggests that patterns of attrition are not wildly different across panels.

Within each panel, we created a dummy variable indicating whether a PID-7 response opportunity was missing and examined its correlation with respondent-level attributes as of the panel’s first survey wave. The TAPS panel presents the best opportunity to discern which respondent-level attributes predict panel attrition because this survey maintained a consistent sampling methodology across waves. Older respondents, Whites, and those with greater income were less likely to exhibit missingness than younger, non-White, and lower-income respondents, respectively. Females were more likely to exhibit missingness than males, as were the least educated compared to the more educated. Many of these correlations were also observed in the ISCAP panel, despite its less consistent sampling procedures.

Although partisan intensity in the first TAPS wave was not associated with missingness over the course of the time series, partisan identity was: Democrats were more likely to miss a response opportunity than Republicans. However, there was no difference in the observed rate of missingness by initial partisan identity in the ISCAP panel. Overall, the demographic and partisan asymmetries in response rates is a concern, but it does not appear to be the case that restricting attention to those who answered all waves zeroes in on especially ardent partisans. Nevertheless, in the analysis that follows, we assess the robustness of the results to different ways of handling missingness.

### Response Variability over Time

We turn now to individual-level patterns of stability. Table 3 shows that respondents tended to give consistent answers to the PID-7 questions at different points in time. For each respondent, we calculated the standard deviation of responses across all waves in which their PID-7 was measured. For example, a respondent classified as a weak Republican in every survey would have a standard deviation of zero.

**Table 3:** Respondent standard deviations among complete and incomplete cases

	ISCAP		VSG		TAPS	
	Complete	Incomplete	Complete	Incomplete	Complete	Incomplete
	(N = 365)	(N = 2241)	(N = 4013)	(N = 5535)	(N = 445)	(N = 2870)
Minimum	0.00	0.00	0.00	0.00	0.00	0.00
25th-percentile	0.00	0.00	0.00	0.00	0.00	0.00
Median	0.33	0.00	0.00	0.00	0.41	0.43
Mean	0.38	0.35	0.36	0.39	0.45	0.48
75th-percentile	0.53	0.58	0.52	0.58	0.64	0.73
Maximum	2.52	3.20	3.10	4.24	2.77	3.21

The PID-7 scale ranges from − 3 (“Strong Democrat”) to $+$ 3 (“Strong Republican”)

In each panel, a notable share of respondents, roughly half, offered identical PID-7 responses over time, irrespective of their status as a complete or “incomplete” case (i.e., respondents who had at least one missing PID-7 value). However, distributions of PID-7 dispersion for incomplete case respondents tended to have longer tails. In each panel, the incomplete case respondents at the 75th and 99th percentile had greater dispersion than their complete case counterparts. Nevertheless, only a small fraction exhibited considerable variation.

**Fig. 2:**
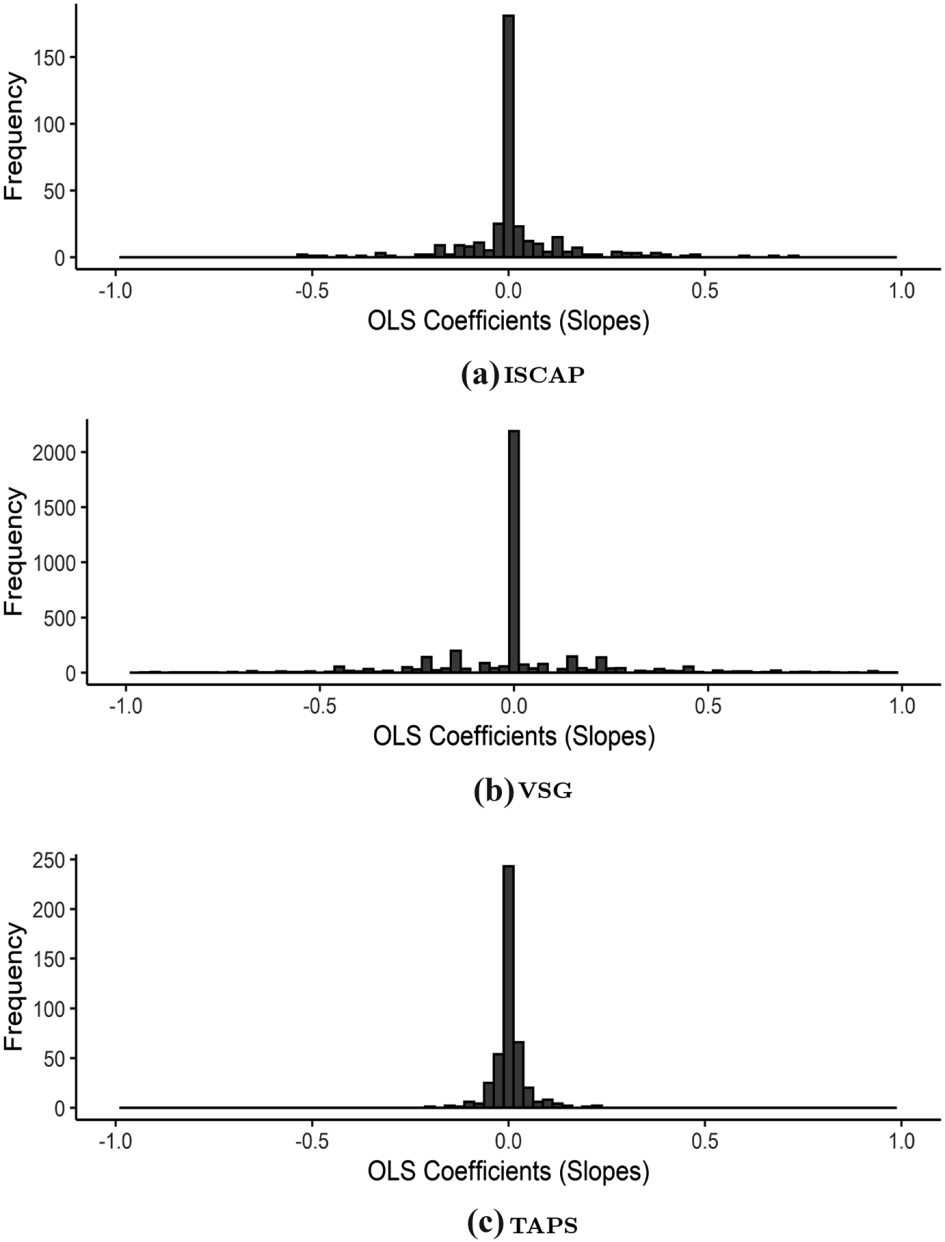
Histograms of complete case respondents’ PID-7 trajectories across each panel’s full time series

Another way to characterize the over-time variation in each individual’s responses is to allow for linear trends. To assess drift in each respondent’s expressed PID-7 over successive waves, we regressed PID-7 values on wave count (enumerated consecutively starting at 0). Figure 2 displays histograms of the coefficient estimates for the wave count regressor for each panel’s set of complete cases. Across each of the three panels, a large plurality of respondents had precisely zero drift; for example, 46.6% of complete case respondents in the ISCAP panel had PID-7 values that were identical in all nine waves. Although a sizeable share of respondents had nonzero coefficients, only 12.3%, 12.5%, and 8.5% of PID-7 response distributions produced coefficients large enough in absolute value to yield a one-point change or greater in PID-7 over the full ISCAP, VSG, and TAPS time series, respectively.^8^

### Correlations over Time

Product-moment correlations offer a rough sense of the stability in response distributions over time. A correlation of 1.0 implies that respondents’ partisanship measured in one wave perfectly predicts their relative position in a subsequent wave. (We say “relative position” because, in principle, the intercept could differ from zero and the slope could differ from one.) Table 4 reports PID-7 correlations using listwise and pairwise deletion of missing data for each panel.^9^ As expected, waves more chronologically proximal to each other produced higher correlations than waves further apart, though the correlation between any pair of waves in any configuration in any panel was never lower than 0.818. For example, in the VSG panel, the listwise correlation between responses recorded in waves 1 and 2 was an impressive 0.946, whereas the listwise correlation between responses recorded in waves 1 and 6, measures taken seven years apart, was still 0.863. The pairwise-deletion correlations for these pairs of waves were nearly identical to their listwise-deletion counterparts: 0.942 and 0.866, respectively.

**Table 4:** Listwise and pairwise correlation matrices: PID-7

	Wave 6	Pre-wave 10	Pre-wave 11	Wave 13	Pre-wave 14	Wave 15
ISCAP						
Wave 6	**	0.947	0.928	0.924	0.900	0.880
Pre-wave 10	0.931	**	0.958	0.941	0.905	0.914
Pre-wave 11	0.919	0.935	**	0.953	0.921	0.914
Wave 13	0.898	0.921	0.933	**	0.956	0.925
Pre-wave 14	0.877	0.898	0.910	0.946	**	0.935
Wave 15	0.871	0.894	0.895	0.915	0.935	**

	Wave 1	Wave 2	Wave 3	Wave 4	Wave 5	Wave 6
VSG						
Wave 1	**	0.946	0.887	0.875	0.872	0.863
Wave 2	0.942	**	0.913	0.899	0.899	0.889
Wave 3	0.883	0.904	**	0.964	0.963	0.951
Wave 4	0.869	0.889	0.957	**	0.966	0.952
Wave 5	0.868	0.890	0.958	0.962	**	0.973
Wave 6	0.866	0.888	0.946	0.949	0.964	**

	Survey 1	Survey 11	Survey 25	Survey 38	Survey 54	Survey 70
TAPS						
Survey 1	**	0.907	0.885	0.873	0.859	0.847
Survey 11	0.888	**	0.932	0.921	0.914	0.901
Survey 25	0.872	0.919	**	0.934	0.918	0.914
Survey 38	0.862	0.904	0.909	**	0.936	0.913
Survey 54	0.848	0.892	0.903	0.905	**	0.947
Survey 70	0.818	0.868	0.884	0.881	0.917	**

Listwise correlations appear in the upper diagonals and pairwise correlations appear in the lower diagonals of each pane. Pairwise correlation cell counts appear in Online Appendix Tables A8, A9, A10, A11 and A12

We compared these patterns to those of two historical four-wave panels previously analyzed in Green and Palmquist (1994), the 1956–1960 and 1980 American National Election Studies panels (see Online Appendix Table A13). We selected these panels because they were nationally representative, they were each four waves long (the largest number of waves in which party identification was measured), they provide evidence from different political moments, and they differed in their average duration between waves. In both panels, over time correlations diminished as distance increased between waves, just as they did in the more recent period. However, wave-to-wave correlations appeared to be slightly higher in the 2011–2020 panels than they were in the earlier panels, even in ANES 1980, where waves were only months, rather than years, apart.

## Modeling

Although raw over-time correlations suggest that party identification is stable by comparison to most other expressions of political opinions, these correlations still suggest that party identification is subject to change. For example, a correlation of 0.95 between two successive survey waves implies that $0.95^2=0.9025$ or 90% of the variance in $wave_t$ is predicted by $wave_{t-1}$. On the other hand, a correlation of 0.98 between two successive survey waves implies that 96% of the variance in $wave_t$ is predicted by $wave_{t-1}$, which suggests a much slower pace of change. In other words, when correlations are high, subtle differences in the magnitude of these correlations make a difference for the characterization of partisan stability. For this reason, methodological choices about whether and how to “disattenuate” observed correlations that are arguably distorted by measurement error can profoundly affect substantive conclusions about the pace of partisan change.

Corrections for measurement error require modeling assumptions. When evaluating these models, it is important to attend both to the plausibility of the assumptions and the statistical consequences of violating them. In this section, we review the assumptions of the most widely used method of correcting for measurement error, which is rooted in instrumental variables (IV) estimation. An alternative approach (Green & Schickler, 1993; Ansolabehere et al., 2008) is to build multi-item scales, which are deemed sufficiently reliable to require no further statistical adjustment. We review the assumptions of both approaches. In the next section, we show that they produce similar results.

### Instrumental Variables Corrections for Measurement Error

**Fig. 3:**
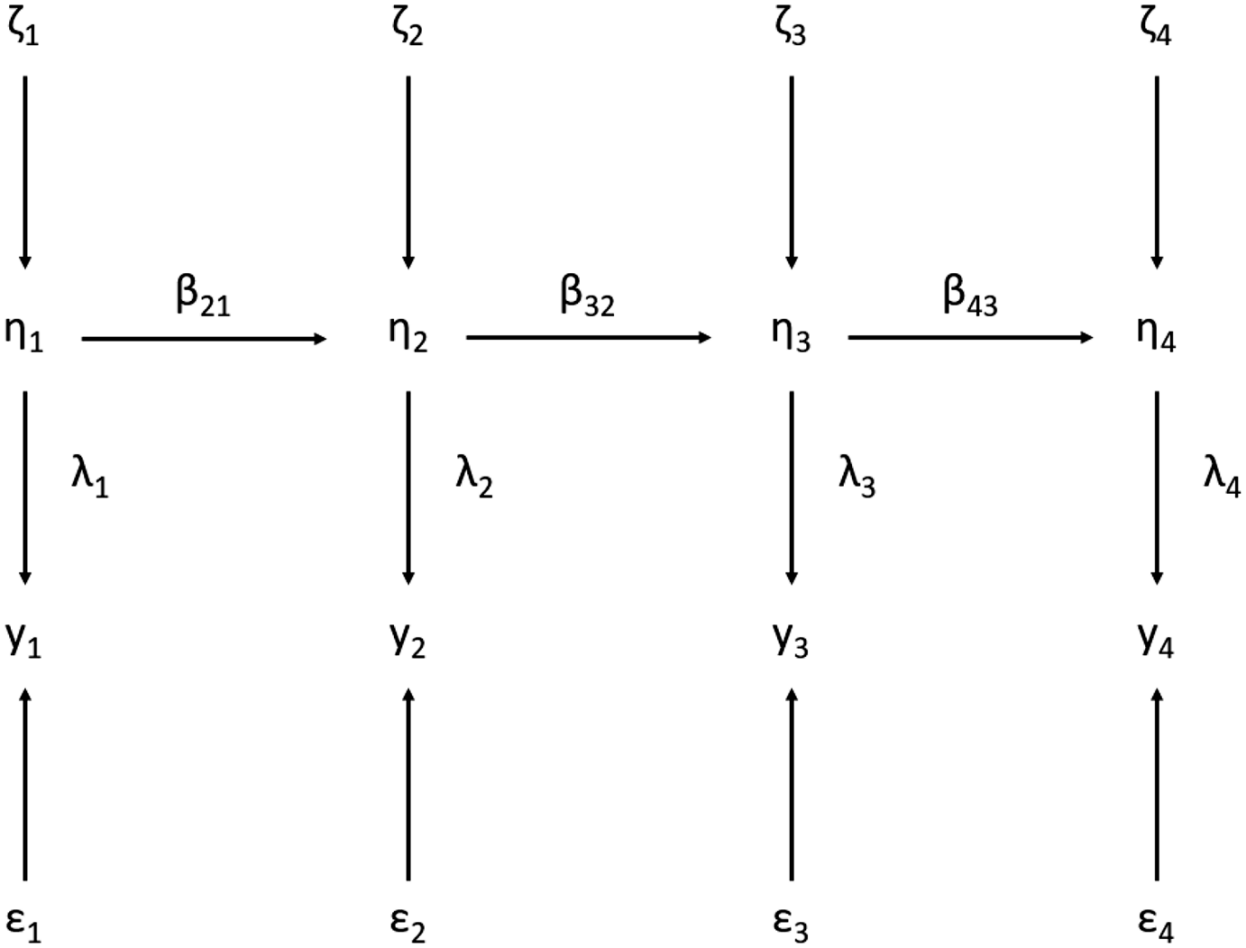
Latent variable model of change with measurement errors. The normalization used in our analysis imposes the restriction that $\lambda _{1}=\lambda _{2}=\lambda _{3}=\lambda _{4}$

Wiley and Wiley (1970) propose an instrumental variables regression model that has become a workhorse for scholars studying the stability of traits measured imperfectly in panel surveys. Their model imagines that a single survey measure is used to gauge a latent trait over at least three waves of data collection (Fig. 3 depicts a four-wave version of their configuration). For each respondent *i* at each period *k*, the latent trait $\eta _{ki}$ is measured, possibly with error $\epsilon _{ki}$. The observed variable at each wave is simply a combination of trait and error:

$$\begin{aligned} y_{ki} = \eta _{ki} + \epsilon _{ki}. \end{aligned}$$

This equation has no intercept when the $y_{ki}$ are re-centered to have mean zero. The evolution of the trait over time is characterized as an autoregressive process plus new disturbance $\zeta _{ki}$. In the first wave:

$$\begin{aligned} \eta _{1i} = \zeta _{1i}. \end{aligned}$$

In the second wave, the trait in the first wave becomes a predictor, and a new disturbance is introduced:

$$\begin{aligned} \eta _{2i} = \beta _{21} \eta _{1i} + \zeta _{2i}. \end{aligned}$$

An analogous equation follows for all subsequent waves. For example:

$$\begin{aligned} \eta _{3i} = \beta _{32} \eta _{2i} + \zeta _{3i}. \end{aligned}$$

Wiley and Wiley (1970) show that, for three waves of panel data, the parameter $\beta _{32}$ and the variance of $\epsilon _{2i}$ can be estimated consistently under the assumption that the disturbances $\zeta _{ki}$ and measurement errors $\epsilon _{ki}$ are all statistically independent. This result is identical to the conventional instrumental variables approach to addressing errors-in-variables bias, using $y_{1i}$ as an instrument for $y_{2i}$ in order to estimate $\beta _{32}$. By comparing the IV estimate of $\beta _{32}$ to the OLS estimate (obtained by simply regressing $y_{3i}$ on $y_{2i}$), one can back out the measurement error variance.

To see the identification strategy that underlies the instrumental variables approach, consider the implications of the above model for the limiting covariance matrix in the absence of sampling error. The covariance between $y_{1i}$ and $y_{2i}$ may be expressed as:

$$\begin{aligned} COV(y_{1i},y_{2i}) = COV(\zeta _{1i}+\epsilon _{1i},\beta _{21} \zeta _{1i}+\zeta _{2i}+ \epsilon _{2i}) = \beta _{21} VAR(\zeta _{1i}). \end{aligned}$$

Similarly, the covariance between $y_{1i}$ and $y_{3i}$ may be expressed as:

$$\begin{aligned} COV(y_{1i},y_{3i}) = COV(\zeta _{1i}+\epsilon _{1i},\beta _{32}\beta _{21} \zeta _{1i}+\beta _{32} \zeta _{2i}+ \epsilon _{3i}) = \beta _{32} \beta _{21} VAR(\zeta _{1i}). \end{aligned}$$

Collecting terms gives the standard instrumental variables identification result:

$$\begin{aligned} {COV(y_{1i},y_{3i}) \over {COV(y_{1i},y_{2i})}} = \beta _{32}. \end{aligned}$$

The same approach can be used to solve for the measurement error variance in the second wave $(VAR(\epsilon _{2i}))$ as well as the variance of the latent trait $(VAR(\eta _{2i}))$.^10^

Wiley and Wiley further assume that the $\epsilon _{ki}$ have equal variance at each wave (as would be the case if measurement error arose mechanically through a persistently flawed measurement process) and that $\beta _{21} = \beta _{32}$ (as might plausibly be the case if waves 1 and 2 were separated by the same amount of time as waves 2 and 3). With these supplementary assumptions, all of the disturbance variances become identified as well.

Some of these restrictions may be relaxed with panels of more than three waves, such as the model depicted in Fig. 3. Just as IV generates consistent estimates of $\beta _{32}$ and $VAR(\epsilon _{2i})$ with three waves of panel data, it also produces consistent estimates of $\beta _{43}$ and $VAR(\epsilon _{3i})$ with four waves of data (Palmquist & Green, 1992). The identification of another measurement parameter $VAR(\epsilon _{3i})$ is especially valuable because it allows one to compute an $R^2$ statistic for wave 2’s prediction of wave 3 that is corrected for measurement error in both of those waves.^11^

What kinds of assumptions make sense in the context of panel data measuring party identification over time? One concern is linearity. The measurement equations presuppose that the observed outcome is trait plus error, but this assumption may be violated for some ranges of the trait, especially given ceiling and floor constraints.

Another concern is whether errors of measurement are correlated from one wave to the next, as might occur if partisan respondents repeatedly exaggerated the extent of their “Independent” identification. For the estimator of $\beta _{32}$, the sign and magnitude of this bias depends on $COV(\epsilon _{1i},\epsilon _{3i})$ in the numerator and $COV(\epsilon _{1i},\epsilon _{2i})$ in the denominator. Absent supplementary measures of party identification in each wave, one cannot validate this assumption empirically. However, the fact that the estimated reliability of PID-7 is similar when measurement models use multiple measures at a given time or a single measure over time suggests that nonrandom errors are a relatively minor concern (Green & Schickler, 1993).

Perhaps the most difficult assumptions to evaluate are those involving the structural model, which assumes that party identification in a given period is affected solely by party identification in the previous period and that the disturbances are independently drawn at each period. It could be the case that disturbances in different periods are related; for example, a person who is undergoing a life change that affects partisanship in wave 1 might still be experiencing new manifestations of this transformation in wave 2. Again, the implications for bias are apparent at an abstract level by rewriting the estimator without assuming that $COV(\zeta _{ki}, \zeta _{ji})$ equals zero. However, it is unclear what one would intuitively expect to see in these covariances if one could somehow calculate them. For this reason, scholars have looked to measurement approaches other than single-indicator panel models for confirmation (e.g., Green & Schickler, 1993). Or they have analyzed a wide assortment of surveys from different periods and countries in order to see whether distinctive wave-to-wave shocks have any appreciable effect on readings of partisan stability (Green & Palmquist, 1994; Schickler & Green, 1997).

One approach that has attracted a good deal of attention is the index-creation strategy popularized by Ansolabehere et al. (2008). Although this approach has the advantage of simplicity and transparency—just add together measures and take their average—it assumes that the resulting index contains no measurement error variance. If the index does contain random error, this method may understate over-time stability. Nevertheless, the indexing approach provides a reasonable robustness check for other methods.

## Results

We begin by considering how models that are prone to errors-in-variables bias compare to instrumental variables models. Table 5 reports the results of OLS regressions in which PID-7 for each wave is regressed on the same measure in the preceding wave.^12^ Across the three panels, OLS slope estimates range from 0.889 to 0.992, and the corresponding $R^2$ values range from 0.79 to 0.98. The implication is that 2% to 20% of the variance in partisanship is “fresh” from wave to wave. Instrumental variables regression reports larger slopes, ranging from 0.93 to 1.01; the reported $R^2$ values, however, are essentially unchanged because IV conventionally reports the squared correlation between the dependent variable and the observed regressor times the estimated slope. Since OLS maximizes the $R^2$, the IV estimates slightly reduce $R^2$. In order to disattenuate the $R^2$ in a manner that accounts for measurement error in both the independent and dependent variables, we apply the Wiley–Wiley estimator for measurement error in waves 2 and 3 of a four-wave panel, as explained in footnote 11. This correction alters the implied rate of partisan change. Focusing on results for the second wave through the fifth wave out of the six selected, we find that the average $R^2$ rises from 0.894 before correction for measurement error to 0.953 after correction for measurement error.^13^

**Table 5:** Estimates of lagged PID-7 effect on PID-7 via OLS regression, IV regression, and Wiley and Wiley (1970) method

ISCAP					
	8/15∼10/12	8/16∼8/15	10/18∼8/16	7/19∼10/18	10/20∼7/19
OLS slope	0.966	0.943	0.974	0.972	0.936
(SE)	(0.017)	(0.015)	(0.016)	(0.016)	(0.019)
Reported R$^{2}$	0.8960	0.9177	0.9085	0.9131	0.8738
IV Slope	N/A	0.965	1.003	0.983	0.969
(SE)	N/A	(0.016)	(0.017)	(0.017)	(0.020)
Reported R$^{2}$	N/A	0.9173	0.9077	0.9129	0.8727
Wiley–Wiley slope*	N/A	0.965	1.003	0.983	N/A
(SE)	N/A	(0.016)	(0.017)	(0.017)	N/A
Implied R$$^{2**}$$	N/A	0.9669	0.9466	0.9562	N/A

VSG					
	11/12∼12/11	12/16∼11/12	7/17∼12/16	4/18∼7/17	12/18∼4/18
OLS slope	0.970	0.903	0.956	0.972	0.992
(SE)	(0.005)	(0.006)	(0.004)	(0.004)	(0.004)
Reported R$^{2}$	0.8941	0.8329	0.9297	0.9329	0.9463
IV Slope	N/A	0.928	0.976	1.005	1.006
(SE)	N/A	(0.007)	(0.005)	(0.004)	(0.004)
Reported R$^{2}$	N/A	0.8323	0.9293	0.9318	0.9461
Wiley–Wiley slope*	N/A	0.928	0.976	1.005	N/A
(SE)	N/A	(0.007)	(0.005)	(0.004)	N/A
Implied R$$^{2**}$$	N/A	0.8743	0.9816	0.9777	N/A

TAPS					
	10/12∼11/11	12/13∼10/12	1/15∼12/13	5/16∼1/15	1/18∼5/16
OLS Slope	0.977	0.889	0.958	0.937	0.943
(SE)	(0.022)	(0.016)	(0.017)	(0.017)	(0.015)
Reported R$^{2}$	0.8222	0.8692	0.8725	0.8767	0.8967
IV slope	N/A	0.930	1.012	0.984	0.970
(SE)	N/A	(0.018)	(0.019)	(0.018)	(0.016)
Reported R$^{2}$	N/A	0.8673	0.8697	0.8745	0.8960
Wiley–Wiley slope*	N/A	0.930	1.012	0.984	N/A
(SE)	N/A	(0.018)	(0.019)	(0.018)	N/A
Implied R$$^{2**}$$	N/A	0.9621	0.9684	0.9475	N/A

The dates displayed correspond to waves 6, 10_pre, 11, 13, 14_pre, and 15 in the ISCAP panel, all six waves in the VSG panel, and surveys 1, 11, 25, 38, 54, and 70 in the TAPS panel

*Wiley–Wiley estimates are based on the three-wave version of the estimator and thus are the same as the IV estimates

**Disattenuated $R^{2}$ values are based on applying the measurement error variance estimator to four-wave panels, as explained in the text

Tables 6, 7, and 8 repeat this analysis, focusing on temporally overlapping sets of survey waves across the three panels. For example, Table 6 showcases the results from four survey waves fielded nearly contemporaneously in both the ISCAP and TAPS panels. Despite the somewhat disparate $R^2$ resulting from the estimated relationship between 2012 and 2015 PID-7 responses before correcting for measurement error (0.882 versus 0.830), ISCAP and TAPS panelists present much more similar—and higher—$R^2$ after the Wiley–Wiley correction is administered (0.948 versus 0.933, respectively). Similarly, the comparison between VSG and TAPS respondents in Table 7 shows converging $R^2$; approximately a four percentage point difference prior to correction (0.824 versus 0.783) was reduced to less than one after both estimates of $R^2$ increased (0.874 versus 0.881, respectively).

**Table 6:** Comparison of estimates of lagged PID-7 effect on PID-7 during overlapping time periods

ISCAP			
	12/12∼10/12	8/15∼12/12	12/16∼8/15
OLS Slope	0.978	0.947	0.952
(SE)	(0.009)	(0.012)	(0.010)
Reported R$^{2}$	0.9417	0.8825	0.9173
IV Slope	N/A	0.978	0.991
(SE)	N/A	(0.013)	(0.011)
Reported R$^{2}$	N/A	0.8816	0.9158
Wiley–Wiley slope*	N/A	0.978	N/A
(SE)	N/A	(0.013)	N/A
Implied R$$^{2**}$$	N/A	0.9479	N/A

TAPS			
	11/12∼10/12	9/15∼11/12	12/16∼9/15
OLS Slope	0.928	0.913	0.934
(SE)	(0.012)	(0.015)	(0.015)
Reported R$^{2}$	0.8827	0.8330	0.8242
IV Slope	N/A	0.971	0.983
(SE)	N/A	(0.016)	(0.017)
Reported R$^{2}$	N/A	0.8296	0.8219
Wiley–Wiley slope*	N/A	0.971	N/A
(SE)	N/A	(0.016)	N/A
Implied R$$^{2**}$$	N/A	0.9327	N/A

ISCAP vs. TAPS

The dates displayed correspond to waves 6, 7, 10_pre, and 12 in the ISCAP panel and surveys 11, 12, 46, and 61 in the TAPS panel

*Wiley–Wiley estimates are based on the three-wave version of the estimator and thus are the same as the IV estimates

**Disattenuated $R^{2}$ values are based on applying the measurement error variance estimator to four-wave panels, as explained in the text

**Fig. 4:**
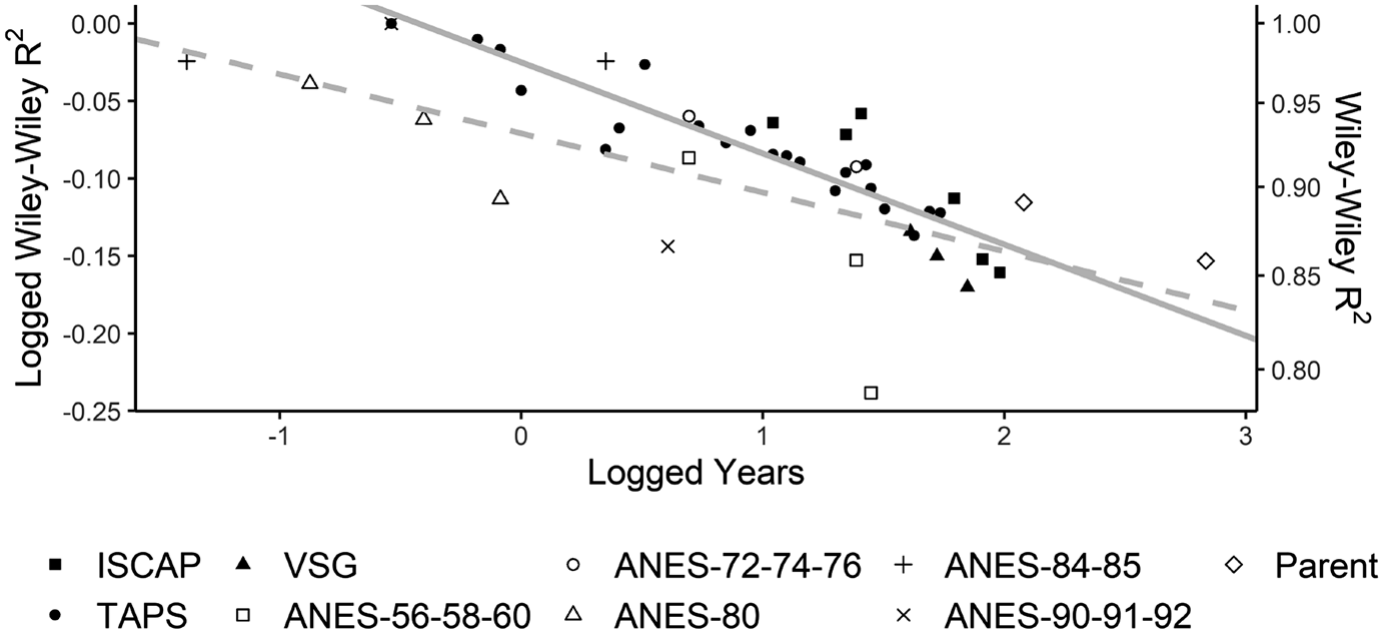
Logged Wiley–Wiley $R^2$ by logged years for three recent and six historical panel surveys. Closed-shaped points represent logged Wiley–Wiley $R^2$ estimates for ISCAP, VSG, and TAPS. Open-shaped points represent logged $R^2$ estimates for six national panels previously analyzed in Green and Palmquist (1994) that collectively spanned 1956–1992. The solid line is a fitted regression line of the estimates associated with the three recent surveys, which has an intercept of − 0.02509 and a slope of − 0.05881. Note that the intercept implies a 1-year $R^2$ estimate of $exp(-0.02509) = 0.975.$ The dashed line is a fitted regression line of the points associated with the six historical panels, which has an intercept of − 0.07092 and a slope of − 0.03813. The implied 1-year $R^2$ is lower, 0.932

To summarize the results, Fig. 4 shows how the passage of time between waves (the horizontal axis, on a log scale) diminishes the disattenuated $R^2$ with which party identification at one point in time predicts party identification in a subsequent interview (the vertical axis, also on a log scale). The solid line shows the fitted regression function using the estimates from the three panel studies, while the dashed line shows the fitted regression function for the estimates of six nationally representative panel surveys fielded between 1956 and 1992, which Green and Palmquist (1994) previously used to characterize the annual rate at which $R^2$ declines.^14^ Although the older data sets generate a more dispersed set of estimates and a somewhat steeper sloping line, an F-test reveals no statistically significant difference between the two lines. If anything, the slightly flatter solid line suggests that the rate of change was even more gradual during the 2011–2020 period.

**Table 7:** Comparison of estimates of lagged PID-7 effect on PID-7 during overlapping time periods

VSG			
	11/12∼12/11	12/16∼11/12	7/17∼12/16
OLS Slope	0.965	0.897	0.953
(SE)	(0.005)	(0.005)	(0.004)
Reported R$^{2}$	0.8874	0.8247	0.9181
IV Slope	N/A	0.926	0.978
(SE)	N/A	(0.006)	(0.004)
Reported R$^{2}$	N/A	0.8238	0.9175
Wiley–Wiley slope*	N/A	0.926	N/A
(SE)	N/A	(0.006)	N/A
Implied R$$^{2**}$$	N/A	0.8744	N/A

TAPS			
	11/12∼11/11	12/16∼11/12	7/17∼12/16
OLS Slope	0.944	0.913	0.935
(SE)	(0.020)	(0.020)	(0.013)
Reported R$^{2}$	0.7912	0.7856	0.8976
IV Slope	N/A	0.967	0.989
(SE)	N/A	(0.022)	(0.015)
Reported R$^{2}$	N/A	0.7829	0.8945
Wiley–Wiley slope*	N/A	0.967	N/A
(SE)	N/A	(0.022)	N/A
Implied R$$^{2**}$$	N/A	0.8807	N/A

VSG vs. TAPS

The dates displayed correspond to waves 1, 2, 3, and 4 in the VSG panel and surveys 1, 12, 61, and 66 in the TAPS panel

*Wiley–Wiley estimates are based on the three-wave version of the estimator and thus are the same as the IV estimates

**Disattenuated $R^{2}$ values are based on applying the measurement error variance estimator to four-wave panels, as explained in the text

**Table 8:** Comparison of estimates of lagged PID-7 effect on PID-7 during overlapping time periods

ISCAP		
	12/16∼12/12	10/18∼12/16
OLS Slope	0.927	0.961
(SE)	(0.015)	(0.013)
Reported R$^{2}$	0.8580	0.9019
IV Slope	N/A	0.997
(SE)	N/A	(0.014)
Reported R$^{2}$	N/A	0.9005
Wiley–Wiley slope	N/A	N/A
(SE)	N/A	N/A
Implied R$^{2}$	N/A	N/A

VSG		
	12/16∼11/12	12/18∼12/16
OLS Slope	0.898	0.963
(SE)	(0.005)	(0.004)
Reported R$^{2}$	0.8308	0.8957
IV Slope	N/A	0.992
(SE)	N/A	(0.005)
Reported R$^{2}$	N/A	0.8949
Wiley–Wiley slope	N/A	N/A
(SE)	N/A	N/A
Implied R$^{2}$	N/A	N/A

ISCAP vs. VSG

The dates displayed correspond to waves 7, 12, and 13 in the ISCAP panel and waves 2, 3, and 6 in the VSG panel

### Addressing Measurement Error by Indexing

We also examine how results change when partisan stability is assessed by pooling measures of party identification over successive surveys using the indexing method. For simplicity, let’s refer to the nine waves in the ISCAP panel by letter, ranging from A through I. We combine waves (A,B,C), waves (D,E,F), and waves (G,H,I) by taking the average score of each triplet. Because each index is more reliable than its component parts, applying OLS and IV should give similar results. In this case, we use the index comprised of waves (D,E,F) as the predictor of the index comprised of waves (G,H,I); for instrumental variables estimation, we use the index comprised of waves (A,B,C) as the instrument. OLS estimates the slope to be 0.990, as compared to 0.984 using IV. The conventional $R^2$ is approximately 0.9335 for both estimation methods. By way of comparison, the OLS estimate of the effect of wave E on wave H is 0.968 with an $R^2$ of 0.8991, and the IV estimate of the effect of wave E on wave H is 0.971 with an $R^2$ of 0.8990. The effect on the $R^2$ is even more pronounced when this method is applied to the TAPS data set, where 24 waves are partitioned into three sets of eight measures. In this configuration, the $R^2$ is 0.963 for both OLS and IV. Evidently, multi-item additive scales expunge enough measurement error to make the choice of estimator inconsequential—both OLS and IV suggest high levels of over-time stability.^15^

### Implications for Long-Term Change

Although wave-to-wave changes tend to be modest, net of measurement error, the estimated $R^2$ statistics do not rule out the possibility that substantial changes occur over much longer periods of time. According to the Social Security Administration, a man who reaches voting age in 2021 is expected to live another 63 years. The corresponding figure for women is 68 years.^16^ This span of time far exceeds the largest representative panel survey, which tracks high school students from 1965 to 1997 (Jennings et al., 2005), but we can simulate long-term partisan change based on the results of the three panel studies at hand.

Consider the implications of the finding that the true $R^2$ between underlying partisanship is 0.975 when interviews are separated by one year. Over a four-year period, the implied $R^2$ remains substantial at 0.904. Over an 8-year period, this $R^2$ falls to 0.817. And over a 63-year male voter’s lifetime, this $R^2$ falls to 0.203. True change is typically negligible over the course of a single election campaign, yet the same statistical model implies that these small adjustments add up between the time that a man enters the electorate at 18 and exits at 81.

## Conclusion

Although much has changed in American politics during the decades since the path-breaking 1956–1960 ANES panel survey, the stability of party identification during the Obama-Trump era looks very much as it did during the Eisenhower Administration or, for that matter, during the eras encompassing Vietnam, Watergate, Stagflation, the Iran Hostage Crisis, the Reagan Ascendancy, the Iran-Contra Scandal, and the Persian Gulf War (Green and Palmquist, 1994).

During the 2011–2020 period, raw correlations between party identification scores measured in successive waves of panel interviews tend to be quite high by comparison to most political attitudes. They are higher still when corrections are made for measurement error. Whether these corrections derive from instrumental variables regression or down-to-earth approaches such as index creation, disattenuated correlations imply that party identification changes at a glacial pace.

The same picture emerges from other ways of describing partisan change statistically. Individual-level response variation is relatively rare across panel waves, a pattern affirmed by other recent studies of multi-wave panel studies, most notably Tucker et al. (2019), who analyze twenty-waves of the TAPS panel from 2011 to 2016. They find that shocks at the individual level dissipate quickly; a shock that moves party identification 0.21 scale points in one wave has an effect of just 0.04 scale points 4 months later and just 0.01 eight months later. When we track individual-level partisan trajectories using all three panel datasets, we too find that a small portion of the public experiences durable change, even in turbulent political times. Nor do we see evidence of aggregate party change, whether we track panel respondents over time or examine independent cross-sectional surveys conducted by the Gallup Poll.

Looking back at the dominant theoretical perspectives that are used to explain change or stability in party identification, it seems that our results underscore the importance of deepening social divides. Our initial hypothesis was two-sided in the sense that the stabilizing effects of growing affective polarization and residential segregation could have been overshadowed by the destabilizing effects of changing party issue positions, the emergence of new issues that divide the parties, and new communication technologies that accentuate those divisions. The fact that party identification seems at least as stable now as it did when the parties were less ideologically distinctive and mercurial vindicates a central argument in Campbell et al. (1960), namely, that party attachment is not primarily driven by ideological affinity. We are quick to concede, however, that this conclusion is not rooted in a direct test of individual-level responsiveness to perceived party stances, a test that presents a host of methodological challenges when using non-experimental panel surveys (Lenz, 2012; Green and Palmquist, 1990).

Although stability over time remains a key feature of American party attachments, we conclude by calling attention to the crucial distinction between slow change and none at all. For those who study elections using cross-sectional survey data, the results presented here are reassuring insofar as they suggest that the pace at which partisanship changes is too slow to be consequential during a given election season. At the same time, the caricature of party identification as an “unmoved mover” creates a host of empirical anomalies that become apparent when researchers track partisan attachments over decades and find substantively large and sustained movements (cf. Kollman and Jackson 2021, Chapter 4). To be empirically sustainable, theoretical accounts must explain why party attachments resist change as well as why meaningful changes do occur over voters’ lifetimes.

## Supplementary Information

Below is the link to the electronic supplementary material.

Supplementary file1 (PDF 194 kb)

## Data Availability

Links to the publicly available data sets and replication code can be found at https://doi.org/10.7910/DVN/F9RDNK.

## Footnotes

1 Panelists were initially recruited by the private market research firm Knowledge Networks, then by GfK, then by Ipsos.

2 Panelists were initially recruited by YouGov for the Cooperative Campaign Analysis Project (CCAP), which formed the basis of the VSG panel beginning in 2016. YouGov continues to interview its panelists.

3 The survey is conducted by the Weidenbaum Center on the Economy, Government, and Public Policy at Washington University in St. Louis. Panelists were initially recruited by Knowledge Networks, then by GfK.

4 The stem question asks, “Generally speaking, do you think of yourself as a...” followed by “Republican,” “Democrat,” and “Independent” response options, and two additional options corresponding to “Another party” and “No preference,” whose wording varied slightly by panel. In order to generate a seven-point scale, respondents were funneled to a second prompt conditional on their response to the first. Respondents who selected “Republican” or “Democrat” at the first prompt were subsequently asked: “Would you call yourself a...” and given the following response options: “Strong Republican” and “Not very strong Republican” (for Republicans) or “Strong Democrat” and “Not very strong Democrat” (for Democrats). Respondents who did not express a “Republican” or “Democratic” affiliation at the first prompt were subsequently asked: “Do you think of yourself as closer to the...” or “Do you lean more toward the...” and given the following response options: “Republican Party” or “Democratic Party.” In contrast to ANES surveys, ISCAP and TAPS respondents were not offered a response option corresponding to “Independent” at the second prompt; respondents were only recorded as purely Independent in the PID-7 measure if they volunteered that they felt close to neither party. As a result, ISCAP and TAPS recorded fewer non-leaning Independents than VSG.

5 Three of the nine instances of PID responses occurred during the months prior to a wave’s fielding, via a separate survey instrument that focused on demographics.

6 TAPS conducted 70 surveys on a near-monthly basis between 2011 and 2018, of which 24 recorded PID-7.

7 For example, see the approximately 50% reduction in the number of respondents between wave 7 and later waves of the ISCAP panel. However, despite the decline in the number of invited respondents in ISCAP’s later waves, the share of invited respondents who completed each wave hovered around 80%.

8 We repeated this analysis using the number of months since the first survey wave as the independent variable instead of wave count. The proportion of PID-7 response distributions that produced coefficients large enough to render a PID-7 change of at least one point over the full time series was 11.2%, 12.0%, and 8.8% in the ISCAP, VSG, and TAPS panels, respectively.

9 Online Appendix Tables A5, A6, and A7 present the full correlation matrices for the ISCAP and TAPS panels.

10 Identification follows from the equality $VAR(\epsilon _{2i}) = VAR(y_{2i}) - COV(y_{2i},y_{3i}) {COV(y_{1i},y_{2i}) \over {COV(y_{1i},y_{3i})}}.$ Since the model implies that $VAR(\epsilon _{2i}) = VAR(y_{2i}) - VAR(\eta _{2i}),$ the variance of the latent trait may be expressed as a function of estimable covariances: $VAR(\eta _{2i}) = COV(y_{2i},y_{3i}){COV(y_{1i},y_{2i}) \over {COV(y_{1i},y_{3i})}}.$

11 The conventional three-wave Wiley–Wiley estimator may also be used for this calculation but relies on the assumption that $VAR(\epsilon _{2i})= VAR(\epsilon _{3i})$. That equality constraint is relaxed with four waves of data. The estimator for the $R^2$ is:

$$\begin{aligned} \hat{R}^{2}_{{\eta _{2},\eta _{3}}} = \frac{\widehat{COV}^2 (y_{2i},y_{3i})}{(\widehat{VAR}(y_{2i})-(\widehat{VAR}(\epsilon _{2i}))((\widehat{VAR}(y_{3i})-(\widehat{VAR}(\epsilon _{3i}))}. \end{aligned}$$

12 See Online Appendix Tables A14 and A15 for full sets of results.

13 Online Appendix Table A15, which analyzes every wave-to-wave change over the duration of the 24-wave TAPS panel, further shows that partisan stability is unrelated to whether respondents were interviewed during the lead-up to an election. When interviews occurred shortly before the 2012 or 2014 elections, stability was found to be quite strong, and the same holds for the immediate aftermath of the 2012, 2014, and 2016 elections.

14 See Online Appendix Fig. A1 for the corresponding results when the three recent panel surveys are weighted. Weighted results show less average stability, although the differences between weighted and unweighted estimates are within the margin of error.

15 We also pooled the four observations in each of the 1956–1960 and 1980 ANES panels into two indices comprised of pairs. Although these panels were too short to perform IV regression using indexed values, we were able to observe higher correlations between index values than among individual point values (as displayed in Online Appendix Table A13). For the 1956–1960 panel, the correlation between the first and second index value was 0.907 listwise and 0.861 pairwise. For the 1980 panel, the analogous correlations were 0.918 and 0.910.

16 https://www.ssa.gov/cgi-bin/longevity.cgi.

## Declarations

### Statements and Declarations

The authors received no support from any organization for the submitted work and played no role in collecting the data used here.
