# Supplementary material for: Partisan Stability During Turbulent Times: Evidence from Three American Panel Surveys
Source: Polit Behav. 2022 Nov 25:1–27. Online ahead of print. doi: 10.1007/s11109-022-09825-y (PMC9702886; doi:10.1007/s11109-022-09825-y)
Supplement: Supplementary file 1 — Supplementary file1 (PDF 194 kb) [file 11109_2022_9825_MOESM1_ESM.pdf]

Online Appendix

Table A1: ISCAP: Wave Field Dates and PID-7 Responses per Wave

	Start Date	End Date	PID Responses
Wave 6:	10/19/2012	10/29/2012	2,258
Wave 7:	11/14/2012	1/29/2013	2,158
Prior to Wave 10*	7/28/2015	9/3/2015	1,382
Prior to Wave 11*:	7/15/2016	8/27/2016	1,158
Wave 12:	11/28/2016	12/7/2016	951
Wave 13:	10/23/2018	11/5/2018	1,007
Prior to Wave 14*:	6/11/2019	8/20/2019	926
Wave 14:	1/24/2020	1/31/2020	926
Wave 15:	10/7/2020	10/22/2020	1,030

*Respondents whose PID response date was not within their wave's start and end dates were recoded as missing.

Table A2: TAPS: Wave Field Dates and PID-7 Responses per Wave		
	Survey Month	PID Responses
Survey 1:	November 2011	1,271
Survey 5:	April 2012	1,533
Survey 7:	June 2012	1,444
Survey 10:	September 2012	1,411
Survey 11:	October 2012	1,417
Survey 12:	November 2012	1,400
Survey 17:	April 2013	1,506
Survey 18:	May 2013	1,478
Survey 20:	July 2013	1,424
Survey 25:	December 2013	1,377
Survey 28:	March 2014	1,559
Survey 31:	June 2014	1,516
Survey 34:	September 2014	1,467
Survey 36:	November 2014	1,428
Survey 38:	January 2015	1,460
Survey 44:	July 2015	1,342
Survey 46:	September 2015	1,317
Survey 50:	January 2016	1,675
Survey 51:	February 2016	1,593
Survey 54:	May 2016	1,555
Survey 61:	December 2016	1,418
Survey 64:	April 2017	1,655
Survey 66:	July 2017	1,619
Survey 70:	January 2018	1,941

PID-7 responses were solicited in 24 out of 70 TAPS surveys between November 2011 and January 2018.

Table A3: ISCAP: PID-7 Wave-level Metrics (Complete Cases: N=365)				
	Start Date	End Date	Mean	Standard Deviation
Wave 6:	10/19/2012	10/29/2012	-0.28	2.31
Wave 7:	11/14/2012	1/29/2013	-0.28	2.32
Pre-wave 10:	7/28/2015	9/3/2015	-0.20	2.36
Pre-wave 11:	7/15/2016	8/27/2016	-0.21	2.33
Wave 12:	11/28/2016	12/7/2016	-0.21	2.35
Wave 13:	10/23/2018	11/5/2018	-0.23	2.38
Pre-wave 14:	6/11/2019	8/20/2019	-0.23	2.42
Wave 14:	1/24/2020	1/31/2020	-0.18	2.40
Wave 15:	10/7/2020	10/22/2020	-0.13	2.42

The PID-7 scale ranges from -3 (“Strong Democrat”) to +3 (“Strong Republican”).

Table A4: TAPS: PID-7 Wave-level Metrics (Complete Cases: N=445)

	Survey Month	Mean	Standard Deviation
Survey 1:	November 2011	-0.13	2.15
Survey 5:	April 2012	-0.10	2.28
Survey 7:	June 2012	-0.14	2.24
Survey 10:	September 2012	-0.18	2.28
Survey 11:	October 2012	-0.12	2.32
Survey 12:	November 2012	-0.18	2.28
Survey 17:	April 2013	-0.17	2.23
Survey 18:	May 2013	-0.18	2.22
Survey 20:	July 2013	-0.13	2.22
Survey 25:	December 2013	-0.13	2.21
Survey 28:	March 2014	-0.16	2.22
Survey 31:	June 2014	-0.14	2.28
Survey 34:	September 2014	-0.12	2.28
Survey 36:	November 2014	-0.07	2.29
Survey 38:	January 2015	-0.06	2.27
Survey 44:	July 2015	-0.14	2.24
Survey 46:	September 2015	-0.10	2.27
Survey 50:	January 2016	-0.11	2.26
Survey 51:	February 2016	-0.10	2.30
Survey 54:	May 2016	-0.11	2.27
Survey 61:	December 2016	-0.11	2.33
Survey 64:	April 2017	-0.09	2.26
Survey 66:	July 2017	-0.10	2.31
Survey 70:	January 2018	-0.08	2.26

The PID-7 scale ranges from -3 ("Strong Democrat") to +3 ("Strong Republican").

Table A5: ISCAP: Listwise and Pairwise Correlation Matrices: PID-7

	Wave 6:	Wave 7:	Pre-wave 10:	Pre-wave 11:	Wave 12:	Wave 13:	Pre-wave 14	Wave 14:	Wave 15:
Wave 6:	**	0.98	0.95	0.93	0.94	0.92	0.90	0.90	0.88
Wave 7:	0.96	**	0.95	0.93	0.94	0.93	0.90	0.90	0.88
Pre-wave 10:	0.93	0.93	**	0.96	0.96	0.94	0.91	0.93	0.91
Pre-wave 11:	0.92	0.91	0.94	**	0.96	0.95	0.92	0.94	0.91
Wave 12:	0.93	0.93	0.95	0.95	**	0.95	0.93	0.95	0.93
Wave 13:	0.90	0.89	0.92	0.93	0.95	**	0.96	0.95	0.93
Pre-wave 14:	0.88	0.88	0.90	0.91	0.93	0.95	**	0.96	0.94
Wave 14:	0.89	0.88	0.92	0.93	0.95	0.94	0.95	**	0.95
Wave 15:	0.87	0.85	0.89	0.90	0.92	0.92	0.94	0.95	**

Listwise correlations appear in the upper diagonal and pairwise correlations appear in the lower diagonal.

Table A6: TAPS: Listwise and Pairwise Correlation Matrices: PID-7

	S1:	S5:	S7:	S10:	S11:	S12:	S17:	S18:	S20:	S25:	S28:	S31:
Survey 1:	**	0.903	0.902	0.908	0.907	0.893	0.888	0.886	0.891	0.885	0.871	0.881
Survey 5:	0.898	**	0.934	0.928	0.930	0.923	0.908	0.909	0.904	0.909	0.896	0.900
Survey 7:	0.902	0.927	**	0.910	0.923	0.927	0.917	0.921	0.912	0.910	0.900	0.901
Survey 10:	0.883	0.901	0.904	**	0.944	0.926	0.907	0.921	0.908	0.922	0.906	0.900
Survey 11:	0.888	0.908	0.917	0.929	**	0.943	0.940	0.945	0.930	0.932	0.924	0.929
Survey 12:	0.884	0.897	0.910	0.916	0.928	**	0.938	0.944	0.932	0.916	0.921	0.922
Survey 17:	0.862	0.890	0.908	0.913	0.927	0.923	**	0.970	0.951	0.929	0.936	0.940
Survey 18:	0.878	0.895	0.906	0.908	0.930	0.918	0.940	**	0.949	0.932	0.938	0.937
Survey 20:	0.865	0.879	0.904	0.894	0.917	0.911	0.924	0.927	**	0.922	0.925	0.938
Survey 25:	0.872	0.889	0.907	0.895	0.919	0.903	0.913	0.917	0.916	**	0.939	0.932
Survey 28:	0.857	0.868	0.888	0.884	0.900	0.901	0.913	0.911	0.917	0.920	**	0.938
Survey 31:	0.867	0.876	0.892	0.887	0.910	0.898	0.912	0.917	0.913	0.919	0.919	**
Survey 34:	0.858	0.872	0.893	0.888	0.899	0.900	0.914	0.922	0.914	0.918	0.919	0.927
Survey 36:	0.868	0.879	0.892	0.888	0.912	0.899	0.922	0.925	0.921	0.917	0.911	0.929
Survey 38:	0.862	0.868	0.880	0.891	0.904	0.895	0.904	0.911	0.917	0.909	0.913	0.911
Survey 44:	0.857	0.874	0.886	0.887	0.904	0.893	0.912	0.921	0.915	0.914	0.913	0.925
Survey 46:	0.858	0.877	0.890	0.886	0.905	0.905	0.915	0.920	0.917	0.915	0.908	0.922
Survey 50:	0.845	0.870	0.878	0.881	0.898	0.884	0.902	0.916	0.906	0.911	0.911	0.919
Survey 51:	0.849	0.874	0.879	0.882	0.895	0.892	0.896	0.917	0.902	0.908	0.896	0.910
Survey 54:	0.848	0.865	0.867	0.877	0.892	0.899	0.898	0.907	0.899	0.903	0.901	0.904
Survey 61:	0.821	0.842	0.839	0.864	0.870	0.865	0.870	0.884	0.877	0.886	0.884	0.895
Survey 64:	0.832	0.849	0.860	0.857	0.873	0.875	0.895	0.896	0.900	0.896	0.898	0.888
Survey 66:	0.845	0.856	0.852	0.872	0.880	0.880	0.889	0.889	0.897	0.886	0.901	0.897
Survey 70:	0.818	0.848	0.854	0.850	0.868	0.866	0.877	0.887	0.882	0.884	0.877	0.883

Listwise correlations appear in the upper diagonal and pairwise correlations appear in the lower diagonal.

Table A7: TAPS: Listwise and Pairwise Correlation Matrices: PID-7

	S34:	S36:	S38:	S44:	S46:	S50:	S51:	S54:	S61:	S64:	S66:	S70:
Survey 1:	0.864	0.872	0.873	0.866	0.870	0.872	0.864	0.859	0.849	0.843	0.857	0.847
Survey 5:	0.894	0.902	0.894	0.890	0.898	0.897	0.895	0.892	0.883	0.876	0.890	0.879
Survey 7:	0.899	0.903	0.884	0.899	0.907	0.896	0.898	0.892	0.873	0.890	0.880	0.891
Survey 10:	0.907	0.901	0.904	0.895	0.902	0.911	0.896	0.904	0.898	0.873	0.902	0.884
Survey 11:	0.913	0.923	0.921	0.920	0.919	0.931	0.917	0.914	0.907	0.895	0.905	0.901
Survey 12:	0.930	0.925	0.902	0.927	0.928	0.921	0.922	0.926	0.907	0.901	0.907	0.908
Survey 17:	0.932	0.941	0.926	0.940	0.943	0.936	0.927	0.933	0.897	0.912	0.911	0.916
Survey 18:	0.934	0.944	0.926	0.937	0.940	0.935	0.932	0.936	0.909	0.920	0.914	0.916
Survey 20:	0.931	0.942	0.936	0.932	0.942	0.933	0.922	0.934	0.902	0.913	0.912	0.905
Survey 25:	0.925	0.931	0.934	0.919	0.925	0.934	0.922	0.918	0.923	0.912	0.920	0.914
Survey 28:	0.934	0.928	0.934	0.928	0.929	0.941	0.933	0.928	0.913	0.915	0.931	0.910
Survey 31:	0.935	0.946	0.937	0.937	0.940	0.938	0.931	0.925	0.923	0.910	0.925	0.908
Survey 34:	**	0.940	0.923	0.939	0.943	0.929	0.932	0.939	0.921	0.919	0.925	0.917
Survey 36:	0.937	**	0.940	0.947	0.949	0.938	0.946	0.936	0.927	0.929	0.921	0.916
Survey 38:	0.915	0.932	**	0.941	0.937	0.949	0.935	0.936	0.935	0.927	0.933	0.913
Survey 44:	0.930	0.931	0.921	**	0.959	0.949	0.953	0.953	0.926	0.925	0.934	0.926
Survey 46:	0.927	0.935	0.922	0.941	**	0.951	0.948	0.951	0.929	0.944	0.940	0.940
Survey 50:	0.921	0.926	0.918	0.934	0.941	**	0.946	0.951	0.932	0.921	0.939	0.921
Survey 51:	0.912	0.924	0.911	0.927	0.934	0.934	**	0.950	0.934	0.940	0.943	0.935
Survey 54:	0.902	0.913	0.905	0.918	0.924	0.924	0.928	**	0.946	0.939	0.946	0.947
Survey 61:	0.900	0.913	0.900	0.903	0.910	0.911	0.916	0.917	**	0.944	0.956	0.950
Survey 64:	0.902	0.905	0.903	0.905	0.921	0.909	0.920	0.919	0.938	**	0.938	0.955
Survey 66:	0.907	0.907	0.900	0.909	0.918	0.918	0.920	0.922	0.940	0.943	**	0.943
Survey 70:	0.888	0.896	0.881	0.896	0.910	0.901	0.913	0.917	0.935	0.928	0.934	**

Listwise correlations appear in the upper diagonal and pairwise correlations appear in the lower diagonal.

Table A8: ISCAP: Pairwise Correlation Cell Counts: PID-7

	Wave 6:	Wave 7:	Pre-wave 10:	Pre-wave 11:	Wave 12:	Wave 13:	Pre-wave 14:	Wave 14:	Wave 15:
Wave 6:	**								
Wave 7:	2,058	**							
Pre-wave 10:	1,201	1,212	**						
Pre-wave 11:	1,020	1,031	1,076	**					
Wave 12:	889	902	863	899	**				
Wave 13:	873	897	836	771	635	**			
Pre-wave 14:	804	813	769	712	592	716	**		
Wave 14:	922	938	812	742	667	742	832	**	
Wave 15:	948	959	838	778	691	751	750	854	**

Pairwise correlations are computed after eliminating rows whose values are missing in one or both of the input values for that particular correlation. As a result, comparisons between different pairs of values use different samples. The maximum pairwise sample size can be found in the wave 7/wave 6 cell, and the minimum pairwise sample size can be found in the pre-wave 14/wave 12 cell.

Table A9: VSG: Pairwise Correlation Cell Counts: PID-7

	Wave 1:	Wave 2:	Wave 3:	Wave 4:	Wave 5:	Wave 6:
Wave 1:	**					
Wave 2:	7,787	**				
Wave 3:	7,780	7,917	**			
Wave 4:	5,806	5,902	5,905	**		
Wave 5:	4,558	4,637	4,643	4,602	**	
Wave 6:	5,767	5,868	5,882	4,926	4,742	**

Pairwise correlations are computed after eliminating rows whose values are missing in one or both of the input values for that particular correlation. As a result, comparisons between different pairs of values use different samples. The maximum pairwise sample size can be found in the wave 3/wave 2 cell, and the minimum pairwise sample size can be found in the wave 5/wave 1 cell.

Table A10: TAPS: Pairwise Correlation Cell Counts: PID-7

	S1:	S5:	S7:	S10:	S11:	S12:	S17:	S18:	S20:	S25:	S28:	S31:
Survey 1:	**											
Survey 5:	1,144	**										
Survey 7:	1,077	1,407	**									
Survey 10:	1,059	1,366	1,337	**								
Survey 11:	1,060	1,373	1,339	1,359	**							
Survey 12:	1,054	1,355	1,324	1,332	1,357	**						
Survey 17:	956	1,241	1,214	1,216	1,230	1,229	**					
Survey 18:	943	1,221	1,197	1,203	1,215	1,211	1,439	**				
Survey 20:	912	1,180	1,163	1,168	1,177	1,176	1,383	1,375	**			
Survey 25:	891	1,145	1,129	1,131	1,143	1,143	1,331	1,327	1,305	**		
Survey 28:	872	1,119	1,093	1,100	1,112	1,108	1,276	1,263	1,257	1,278	**	
Survey 31:	847	1,086	1,066	1,065	1,077	1,073	1,241	1,227	1,222	1,239	1,473	**
Survey 34:	831	1,058	1,039	1,039	1,050	1,050	1,204	1,195	1,190	1,203	1,426	1,423
Survey 36:	817	1,036	1,013	1,016	1,026	1,026	1,179	1,168	1,165	1,178	1,389	1,379
Survey 38:	829	1,048	1,027	1,031	1,040	1,041	1,190	1,181	1,180	1,175	1,362	1,355
Survey 44:	782	978	958	964	974	975	1,114	1,107	1,106	1,103	1,272	1,268
Survey 46:	759	956	936	939	946	951	1,089	1,084	1,082	1,082	1,250	1,247
Survey 50:	793	1,003	979	984	990	990	1,135	1,122	1,118	1,121	1,292	1,291
Survey 51:	757	958	938	937	946	947	1,090	1,081	1,076	1,076	1,246	1,248
Survey 54:	751	937	918	920	927	932	1,066	1,057	1,055	1,054	1,216	1,219
Survey 61:	685	861	844	844	851	858	984	978	972	969	1,124	1,123
Survey 64:	631	788	769	772	774	784	904	898	892	893	1,027	1,031
Survey 66:	625	775	760	760	765	774	892	885	881	885	1,012	1,014
Survey 70:	630	788	774	774	779	784	896	888	885	889	1,019	1,022

Pairwise correlations are computed after eliminating rows whose values are missing in one or both of the input values for that particular correlation. As a result, comparisons between different pairs of values use different samples. The maximum pairwise sample size can be found in the S51/S50 cell, and the minimum pairwise sample size can be found in the S66/S1 cell.

Table A11: TAPS: Pairwise Correlation Cell Counts: PID-7

	S34:	S36:	S38:	S44:	S46:	S50:	S51:	S54:	S61:	S64:	S66:	S70:
Survey 1:												
Survey 5:												
Survey 7:												
Survey 10:												
Survey 11:												
Survey 12:												
Survey 17:												
Survey 18:												
Survey 20:												
Survey 25:												
Survey 28:												
Survey 31:												
Survey 34:	**											
Survey 36:	1,373	**										
Survey 38:	1,348	1,333	**									
Survey 44:	1,264	1,259	1,329	**								
Survey 46:	1,245	1,237	1,306	1,279	**							
Survey 50:	1,273	1,257	1,287	1,254	1,239	**						
Survey 51:	1,234	1,216	1,247	1,223	1,213	1,581	**					
Survey 54:	1,206	1,191	1,210	1,186	1,183	1,545	1,513	**				
Survey 61:	1,117	1,101	1,125	1,112	1,104	1,408	1,388	1,368	**			
Survey 64:	1,020	1,011	1,035	1,023	1,016	1,272	1,256	1,253	1,242	**		
Survey 66:	1,006	999	1,021	1,009	1,006	1,244	1,230	1,226	1,218	1,551	**	
Survey 70:	1,011	1,003	1,025	1,014	1,007	1,246	1,230	1,221	1,211	1,454	1,462	**

Pairwise correlations are computed after eliminating rows whose values are missing in one or both of the input values for that particular correlation. As a result, comparisons between different pairs of values use different samples. The maximum pairwise sample size can be found in the S51/S50 cell, and the minimum pairwise sample size can be found in the S66/S1 cell.

Table A12: Pairwise Correlation Cell Counts: PID-7

ISCAP						
	Wave 6:	Pre-wave 10:	Pre-wave 11:	Wave 13:	Pre-wave 14:	Wave 15:
Wave 6:	**					
Pre-wave 10:	1,201	**				
Pre-wave 11:	1,020	1,076	**			
Wave 13:	873	836	771	**		
Pre-wave 14:	804	769	712	716	**	
Wave 15:	948	838	778	751	750	**

VSG						
	Wave 1:	Wave 2:	Wave 3:	Wave 4:	Wave 5:	Wave 6:
Wave 1:	**					
Wave 2:	7,787	**				
Wave 3:	7,780	7,917	**			
Wave 4:	5,806	5,902	5,905	**		
Wave 5:	4,558	4,637	4,643	4,602	**	
Wave 6:	5,767	5,868	5,882	4,926	4,742	**

TAPS						
	Survey 1:	Survey 11:	Survey 25:	Survey 38:	Survey 54:	Survey 70:
Survey 1:	**					
Survey 11:	1,060	**				
Survey 25:	891	1,143	**			
Survey 38:	829	1,040	1,175	**		
Survey 54:	751	927	1,054	1,210	**	
Survey 70:	630	779	889	1,025	1,221	**

Pairwise correlations are computed after eliminating rows whose values are missing in one or both of the input values for that particular correlation. As a result, comparisons between different pairs of values use different samples.

Table A13: Listwise and Pairwise Correlation Matrices: PID-7

	1956:	1958:	Sep 1960:	Dec 1960:
**ANES 1956-60**				
1956:	**	0.863	0.846	0.822
1958:	0.853	**	0.890	0.855
Sep 1960:	0.825	0.861	**	0.899
Dec 1960:	0.809	0.826	0.878	**
**ANES 1980**				
	Feb 1980:	Jun 1980:	Sep 1980:	Nov 1980:
Feb 1980:	**	0.853	0.862	0.831
Jun 1980:	0.850	**	0.878	0.863
Sep 1980:	0.862	0.880	**	0.887
Nov 1980:	0.825	0.855	0.887	**

Listwise correlations appear in the upper diagonals and pairwise correlations appear in the lower diagonals of each pane.

Table A14: ISCAP: Estimates of Lagged PID-7 Effect on PID-7 via OLS Regression, IV Regression, and Wiley and Wiley (1970) Estimator

	12/12~10/12	8/15~12/12	8/16~8/15	12/16~8/16	10/18~12/16	7/19~10/18	1/20~7/19	10/20~1/20
OLS Slope	0.979	0.963	0.943	0.971	0.958	0.972	0.953	0.959
(Standard Error)	(.011)	(.018)	(.015)	(.015)	(.017)	(.016)	(.014)	(.017)
Reported R^2	0.9549	0.8924	0.9180	0.9246	0.8958	0.9133	0.9248	0.9017
IV Slope	N/A	0.987	0.967	1.014	1.003	1.000	0.984	0.982
(Standard Error)	N/A	(.018)	(.016)	(.015)	(.018)	(.017)	(.015)	(.017)
Reported R^2	N/A	0.8918	0.9174	0.9228	0.8937	0.9126	0.9238	0.9011
Wiley-Wiley Slope*	N/A	0.987	0.967	1.014	1.003	1.000	0.984	N/A
(Standard Error)	N/A	(.018)	(.016)	(.015)	(.018)	(.017)	(.015)	N/A
Implied R^{2**}	N/A	0.9380	0.9826	1.0000	0.9651	0.9699	0.9777	N/A

The dates displayed correspond to all nine waves in the ISCAP panel.
*Wiley-Wiley estimates are based on the three-wave version of the estimator and thus are the same as the IV estimates.
**Disattenuated R^2 values are based on applying the measurement error variance estimator to four-wave panels, as explained in the text.

Table A15: TAPS: Estimates of Lagged PID-7 Effect on PID-7 via OLS Regression, IV Regression, and Wiley and Wiley (1970) method

	S5∼S1	S7∼S5	S10∼S7	S11∼S10	S12∼S11	S17∼S12	S18∼S17
OLS Slope	0.957	0.917	0.927	0.960	0.926	0.917	0.968
(Standard Error)	(0.022)	(0.017)	(0.020)	(0.016)	(0.015)	(0.016)	(0.012)
Reported R^2	0.8150	0.8725	0.8270	0.8915	0.8897	0.8798	0.9402
IV Slope	N/A	0.980	1.011	1.031	0.963	0.974	1.004
(Standard Error)	N/A	(0.019)	(0.022)	(0.018)	(0.017)	(0.017)	(0.012)
Reported R^2	N/A	0.8683	0.8201	0.8866	0.8883	0.8764	0.9389
Wiley-Wiley Slope*	N/A	0.980	1.011	1.031	0.963	0.974	1.004
(Standard Error)	N/A	(0.019)	(0.022)	(0.018)	(0.017)	(0.017)	(0.012)
Implied R^{2**}	N/A	1.0181	0.9701	0.9956	0.9826	0.9706	1.0095

	S20∼S18	S25∼S20	S28∼S25	S31∼S28	S34∼S31	S36∼S34	S38∼S36
OLS Slope	0.949	0.917	0.943	0.962	0.937	0.945	0.929
(Standard Error)	(0.015)	(0.018)	(0.016)	(0.017)	(0.017)	(0.016)	(0.016)
Reported R^2	0.9002	0.8494	0.8812	0.8799	0.8744	0.8833	0.8835
IV Slope	0.981	0.978	1.008	1.018	0.998	1.017	0.970
(Standard Error)	(0.016)	(0.020)	(0.018)	(0.018)	(0.018)	(0.018)	(0.017)
Reported R^2	0.8991	0.8457	0.8770	0.8769	0.8707	0.8782	0.8817
Wiley-Wiley Slope*	0.981	0.978	1.008	1.018	0.998	1.017	0.970
(Standard Error)	(0.016)	(0.020)	(0.018)	(0.018)	(0.018)	(0.018)	(0.017)
Implied R^{2**}	0.9924	0.9679	0.9970	0.9917	1.0017	0.9931	0.9876

	S44∼S38	S46∼S44	S50∼S46	S51∼S50	S54∼S51	S61∼S54	S64∼S61
OLS Slope	0.929	0.973	0.947	0.964	0.937	0.971	0.919
(Standard Error)	(0.016)	(0.014)	(0.015)	(0.016)	(0.015)	(0.016)	(0.015)
Reported R^2	0.8856	0.9191	0.9050	0.8954	0.9019	0.8945	0.8917
IV Slope	0.994	1.010	0.985	1.015	0.991	1.009	0.965
(Standard Error)	(0.017)	(0.015)	(0.015)	(0.017)	(0.016)	(0.017)	(0.016)
Reported R^2	0.8812	0.9178	0.9035	0.8929	0.8988	0.8931	0.8894
Wiley-Wiley Slope*	0.994	1.010	0.985	1.015	0.991	1.009	0.965
(Standard Error)	(0.017)	(0.015)	(0.015)	(0.017)	(0.016)	(0.017)	(0.016)
Implied R^{2**}	0.9839	0.9931	0.9914	0.9972	0.9922	0.9776	1.0109

	S66∼S64	S70∼S66
OLS Slope	0.958	0.921
(Standard Error)	(0.017)	(0.015)
Reported R^2	0.8805	0.8893
IV Slope	1.034	0.994
(Standard Error)	(0.018)	(0.017)
Reported R^2	0.8750	0.8836
Wiley-Wiley Slope*	1.034	N/A
(Standard Error)	(0.018)	N/A
Implied R^{2**}	1.0254	N/A

The dates displayed correspond to all 24 waves in the TAPS panel.

*Wiley-Wiley estimates are based on the three-wave version of the estimator and thus are the same as the IV estimates.

**Disattenuated R^2 values are based on applying the measurement error variance estimator to four-wave panels, as explained in the text.

Table A16: Estimates of Lagged PID-7 Effect on PID-7 via IV Regression, Unweighted Versus Weighted

**ISCAP**					
	8/15∼10/12	8/16∼8/15	10/18∼8/16	7/19∼10/18	10/20∼7/19
Unweighted IV Slope	N/A	0.965	1.003	0.983	0.969
(Standard Error)	N/A	(0.016)	(0.017)	(0.017)	(0.020)
Reported R^2	N/A	0.9173	0.9077	0.9129	0.8727
Weighted IV Slope	N/A	0.966	0.999	0.967	0.988
(Standard Error)	N/A	(0.016)	(0.018)	(0.017)	(0.017)
Reported R^2	N/A	0.9136	0.8973	0.9085	0.9008
**VSG**					
	11/12∼12/11	12/16∼11/12	7/17∼12/16	4/18∼7/17	12/18∼4/18
Unweighted IV Slope	N/A	0.928	0.976	1.005	1.006
(Standard Error)	N/A	(0.007)	(0.005)	(0.004)	(0.004)
Reported R^2	N/A	0.8323	0.9293	0.9318	0.9461
Weighted IV Slope	N/A	0.935	0.969	1.004	1.006
(Standard Error)	N/A	(0.008)	(0.005)	(0.005)	(0.004)
Reported R^2	N/A	0.7974	0.9258	0.9234	0.9468
**TAPS**					
	10/12∼11/11	12/13∼10/12	1/15∼12/13	5/16∼1/15	1/18∼5/16
Unweighted IV Slope	N/A	0.930	1.012	0.984	0.970
(Standard Error)	N/A	(0.018)	(0.019)	(0.018)	(0.016)
Reported R^2	N/A	0.8673	0.8697	0.8745	0.8960
Weighted IV Slope	N/A	0.912	1.013	0.963	0.954
(Standard Error)	N/A	(0.019)	(0.020)	(0.024)	(0.017)
Reported R^2	N/A	0.8557	0.8525	0.7953	0.8977

The dates displayed correspond to waves 6, 10_pre, 11, 13, 14_pre, and 15 in the ISCAP panel, all six waves in the VSG panel, and surveys 1, 11, 25, 38, 54, and 70 in the TAPS panel.

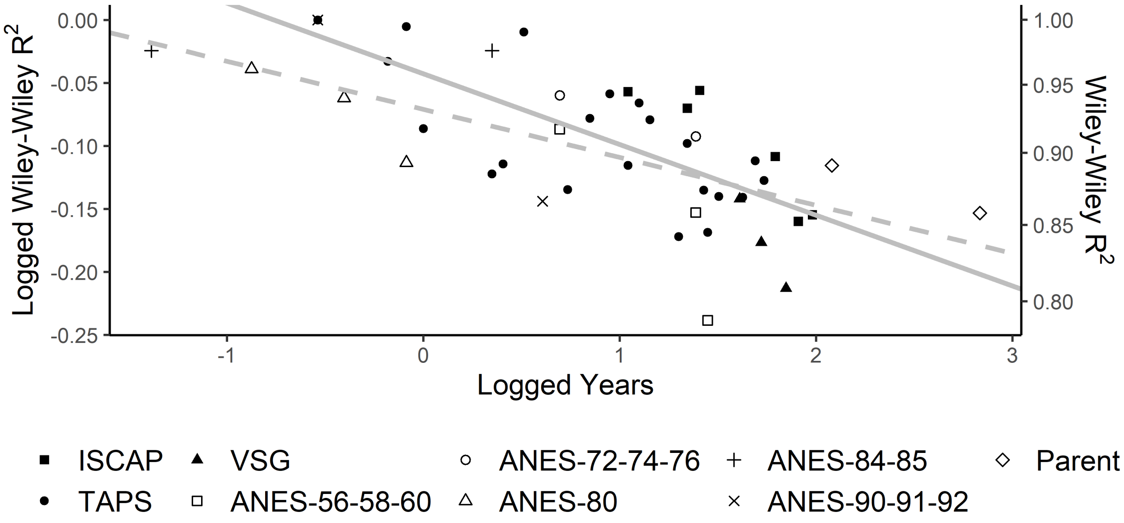

Figure A1: Weighted logged Wiley-Wiley R^2 by logged years for nine panel surveys.
Note: Closed-shaped data points represent logged weighted Wiley-Wiley R^2 estimates for ISCAP, VSG, and TAPS. Open-shaped data points represent logged R^2 estimates for the remaining panels. As in Figure 4, the solid line is a fitted regression line of the estimates associated with the three recent surveys. The weighted estimates produces an intercept of -0.04272 and a slope of -0.05613, which implies a one-year R^2 estimate of $exp(-0.04272) = 0.958$ – compare this to an intercept and slope of -0.02509 and -0.05881 and an implied one-year R^2 of 0.975 for the fitted regression line among unweighted estimates. A one-year R^2 of 0.958 implies a four-year R^2 of 0.842, an eight-year R^2 of 0.709, and a 63-year R^2 of 0.067. The dashed line is the same fitted regression line of the points associated with the six historical panels that appears in Figure 4, which has an implied one-year R^2 of 0.932.
